# MMCL-CDR: enhancing cancer drug response prediction with multi-omics and morphology images contrastive representation learning

**DOI:** 10.1093/bioinformatics/btad734

**Published:** 2023-12-09

**Authors:** Yang Li, Zihou Guo, Xin Gao, Guohua Wang

**Affiliations:** College of Computer and Control Engineering, Northeast Forestry University, Harbin 150006, China; College of Computer and Control Engineering, Northeast Forestry University, Harbin 150006, China; Computational Bioscience Research Center, King Abdullah University of Science and Technology (KAUST), Thuwal, Saudi Arabia; Computer Science Program, Computer, Electrical and Mathematical Sciences and Engineering Division, King Abdullah University of Science and Technology (KAUST), Thuwal, Saudi Arabia; College of Computer and Control Engineering, Northeast Forestry University, Harbin 150006, China

## Abstract

**Motivation:**

Cancer is a complex disease that results in a significant number of global fatalities. Treatment strategies can vary among patients, even if they have the same type of cancer. The application of precision medicine in cancer shows promise for treating different types of cancer, reducing healthcare expenses, and improving recovery rates. To achieve personalized cancer treatment, machine learning models have been developed to predict drug responses based on tumor and drug characteristics. However, current studies either focus on constructing homogeneous networks from single data source or heterogeneous networks from multiomics data. While multiomics data have shown potential in predicting drug responses in cancer cell lines, there is still a lack of research that effectively utilizes insights from different modalities. Furthermore, effectively utilizing the multimodal knowledge of cancer cell lines poses a challenge due to the heterogeneity inherent in these modalities.

**Results:**

To address these challenges, we introduce MMCL-CDR (Multimodal Contrastive Learning for Cancer Drug Responses), a multimodal approach for cancer drug response prediction that integrates copy number variation, gene expression, morphology images of cell lines, and chemical structure of drugs. The objective of MMCL-CDR is to align cancer cell lines across different data modalities by learning cell line representations from omic and image data, and combined with structural drug representations to enhance the prediction of cancer drug responses (CDR). We have carried out comprehensive experiments and show that our model significantly outperforms other state-of-the-art methods in CDR prediction. The experimental results also prove that the model can learn more accurate cell line representation by integrating multiomics and morphological data from cell lines, thereby improving the accuracy of CDR prediction. In addition, the ablation study and qualitative analysis also confirm the effectiveness of each part of our proposed model. Last but not least, MMCL-CDR opens up a new dimension for cancer drug response prediction through multimodal contrastive learning, pioneering a novel approach that integrates multiomics and multimodal drug and cell line modeling.

**Availability and implementation:**

MMCL-CDR is available at https://github.com/catly/MMCL-CDR.

## 1 Introduction

Cancer is a complicated and intractable worldwide disease, and it is also the main cause of death, posing a great threat to human health. Cultured cancer cell lines, characterized by different genomic backgrounds and gene expressions, play an important role in studying drug sensitivity. Although they are different from the genomes of original tissues or tumor samples, cancer cell lines are the basic resources for finding new anticancer drugs in cancer biology. They have also made great contributions to exploring the molecular basis of cancer treatment and determining new anti-cancer treatment methods in the field of precision medicine (Weinstein 2012). Therefore, predicting the response of drug cell lines can help to make treatment plans, improve the treatment effect and reduce the drug cost (Adam *et al.* 2020, Xia *et al.* 2022, Liu and Zhang 2023).

The practice of verifying the drug sensitivity of compounds using genomic information from cell lines *in vitro* can be traced back to the late 1980s. The US National Cancer Institute (NCI) conducted drug screening on over 60 human tumor cell lines, aiming to identify potential anticancer compounds. Subsequently, the NCI60 (Gholami *et al.* 2013) has been consistently employed for investigating the mechanisms underlying growth inhibition and cell death in tumor cell lines. In recent years, several large-scale cell line drug response databases, such as the Cancer Cell Line Encyclopedia (CCLE) (Barretina *et al.* 2012) and the Genomics of Drug Sensitivity in Cancer (GDSC) (Yang *et al.* 2012), have been made accessible to the public. These databases generate substantial drug sensitivity data for thousands of cancer cell lines.

Cancer drug response prediction algorithms can be categorized into three main groups: regression-based, classification-based, and graph-based methods. Regression-based methods treat the prediction of cell-drug responses as a regression task, aiming to predict continuous values like half-maximal inhibitory concentration (IC50) using models such as ridge regression (Geeleher *et al.* 2014) and LASSO (Tibshirani 2018). Classification-based methods can be divided into two categories: traditional machine learning and deep learning methods. Traditional approaches included feature extraction from drugs and cell lines, followed by classification using classifiers like Support Vector Machines (Huang *et al.* 2017, Wang *et al.* 2022) and Random Forest (Su *et al.* 2019). In contrast, deep learning methods use deep neural networks to directly obtain representations of drugs and cell lines and perform classification based on these learned representations. Rampášek *et al.* (2019) introduced the Drug Response Variational Autoencoder, a generative model that jointly learned a drug response predictor and drug perturbation effects in a low-dimensional latent representation of gene expression. Graph-based methods model drug response prediction as a link prediction problem. Building on the idea that drugs with similar chemical structures may produce similar biological effects in known cell lines, Zhang *et al.* (2018) created a heterogeneous network comprising cell lines, drugs, target genes, and their connections to predict drug responses in cell lines using this network. Wang *et al.* (2017) employed a similarity-regularized matrix factorization approach, which seamlessly integrated the chemical structural similarity of drugs and the gene expression profile similarity among cell lines into the matrix factorization model for drug response prediction.

Subsequently, research on predicting drug responses in cell lines has progressed from analyzing single-omic data, such as gene expression, as shown in prior studies (Bussey *et al.* 2006, Choy *et al.* 2008, Januchowski *et al.* 2013, Liu *et al.* 2022b), to jointly modeling and analyzing multiomics data such as gene expression, copy number variation, and DNA methylation in cell lines (Hasin *et al.* 2017, Ali *et al.* 2018, Celebi *et al.* 2019). In addition to gene expression, Sharifi-Noghabi *et al.* (2019) incorporated somatic mutations and copy number variation, and utilized encoders with triplet loss and cross-entropy loss functions to learn features of each omics type, facilitating the prediction of cell line responses. Hira *et al.* (2021) proposed using the Variational Autoencoder (An and Cho 2015) to analyze single-omics, integrated bi-omics, and tri-omics data in the context of ovarian cancer, addressing the challenges posed by high-dimensional multiomics data. Peng *et al.* (2021) integrated multiomics information from cell lines, along with a drug similarity matrix, as input for Graph Convolutional Networks (GCN) (Kipf and Welling 2017) to extract potential features related to cancer cell lines and drugs. Subsequently, they utilized linear correlation coefficients to predict drug responses in cell lines.

Although the multiomics data show potential in predicting drug responses of cancer cell lines, there is still a lack of research to effectively use data from different modalities. Recently, multimodal data have been proved to be effective in various research tasks related to cancer cells (Hajitou *et al.* 2008, Isherwood *et al.* 2011, Andrews *et al.* 2022). Giedt *et al.* (2016) developed an image-based analysis technique to study mitochondrial morphology in cells, revealing the role of mitochondria in the biology and drug response of cancer cells. Based on these findings, they proposed that image-based mitochondrial phenotypes could serve as biomarkers for assessing cancer phenotype and drug response.

Inspired by the mentioned study, this article explores the integration of copy number variation, gene expression, and cell morphology images through multimodal contrastive learning to evaluate the effectiveness of aligning different modalities in cell line representation learning. We introduce a model named MMCL-CDR (Multimodal Contrastive Learning for Cancer Drug Responses) for predicting cancer drug responses (CDR) using multiomics data and morphology images through contrastive learning. We begin by obtaining and preprocessing multimodal data, which includes copy number variation, gene expression, and cell line morphological images, from GDSC (Yang *et al.* 2012) and DMSZ (Parte *et al.* 2020). Additionally, we collect the SMILES strings of drugs from PubChem to construct drug molecular graphs. Next, we employ two distinct encoders to learn representations of copy number variation and gene expression. These representations are then fused by a projection layer with attention mechanism. Simultaneously, a convolutional neural network is applied as image encoder to learn cell line image representation. Subsequently, we apply contrastive learning to align omics and image representations of cell lines, resulting in the concatenation of these two representations to establish the cell line representation. Moreover, we implement a two-layer GCN to aggregate the drug molecular graph, generating the final drug representation. Finally, for predicting cell line drug responses, a multilayer perceptron (MLP) is utilized, leveraging both the cell line and drug representations. We compare our model with the state-of-the-art methods on the constructed dataset. The experimental results clearly indicate that our model outperforms the baselines. Furthermore, the ablation experiments demonstrate that MMCL-CDR successfully integrates histological data and cell line morphological images, resulting in enhanced accuracy in drug response prediction. We also observe that MMCL-CDR effectively clusters similar cell lines by visualizing the cell line representations. These findings are of great significance in revealing the hidden relationships between cancer cell lines and drugs. It is worth noting that, this study introduces a novel approach to multiomics and multimodal cell line representation learning, which carries substantial importance for drug response prediction, advancing cancer treatment, and ultimately improving patient outcomes.

## 2 Materials and methods

**Figure 1.**
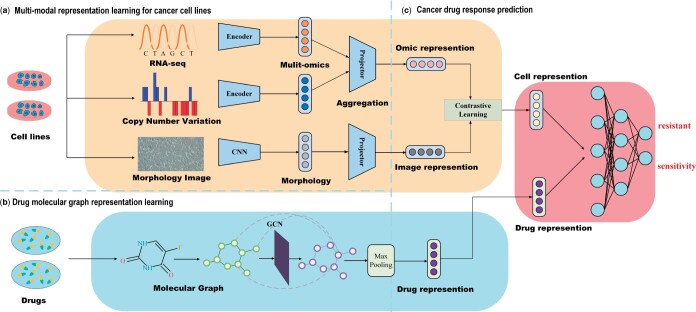
The architecture of multiomics and morphology images with contrastive learning for cancer drug response (MMCL-CDR).

In this section, we introduce the proposed cancer drug response prediction model. As shown in Fig. 1, the overall model consists of three components: multimodal representation learning for cancer cell lines, drug molecular graph representation and CDR prediction. First, we use different encoders to process multiomics data and cell line morphology images, capturing their unique characteristics and obtaining multimodal cell line representations. Second, the drug molecular graph is input into a graph convolutional neural network to learn the drug’s representation. Finally, we aggregate multiple representations of the learned cell lines using an attention mechanism and contrastive learning. These aggregated representations are then input into an MLP along with drug representations for predicting CDR.

### 2.1 Problem definition

We start by defining the task of predicting CDR. To represent the drug response of cancer cell lines, we obtain log-normalized half-maximal inhibitory concentrations (${\text{IC}}_{50}$) values for 22 490 cell line-drug pairs from GDSC. Subsequently, we categorize the ${\text{IC}}_{50}$ values using a threshold based on the reported maximum screening concentration, following previous work (Stanfield *et al.* 2017, Ahmadi Moughari and Eslahchi 2021). This categorization allows us to binarize the association between cell lines and drug responses into “sensitive” and “resistant,” which can be defined as follows:

(1) $$A_{ij}=\{\begin{array}{c} 1\;{\;\text{response}}_{ij}\leq {\;\text{threshold}}_{j}\\ 0\;\;\text{other} \end{array},$$

where ${\text{response}}_{ij}$ is the ${\text{IC}}_{50}$ value between the *i*-th cell line and the *j*-th drug, and the ${\text{threshold}}_{j}$ represents the sensitivity threshold of the *j*-th drug. Finally, we obtain 7809 sensitive pairs and 14 681 resistant pairs, constructed by 254 cell lines and 311 drugs.

### 2.2 Multimodal representation learning for cancer cell lines

Prior research has demonstrated that integrating multiomics data from cell lines can enhance the accuracy of cancer drug response (CDR) prediction (Malik *et al.* 2021, Pu *et al.* 2022). To accomplish this, we initially aggregate multiomics data from GDSC, which is a database focusing on Genomics of Drug Sensitivity in Cancer. The GDSC database comprises data from approximately 75 000 experiments, detailing the responses of around 200 anticancer drugs across more than 1000 different types of tumor cells.

#### 2.2.1 Multiomic feature representation

We download two types of omics data, specifically gene expression and copy number variation data, for a total of 254 cell lines. These 254 cancer cell lines encompass a total of 13 different types of cancer, such as skin cancer, digestive system cancer, blood cancer, and lung cancer. These datasets are used to compute comprehensive omics representations of the cell lines. To process the gene expression values from the GDSC dataset, we convert them into log-normalized TPM (Transcripts Per Million) values. Furthermore, any missing values in both the gene expression and copy number variation datasets are filled with zeros. Subsequently, we apply Gaussian regularization to normalize the gene expression data:

(2) $$\bar{\;\text{exp}\;r_{j}}=\frac{\left(\text{exp}\;r_{j}-\mu_{j}\right)}{\sigma_{j}},$$

where $\text{exp}\;r_{j}$ is the feature representation of gene expression for the *j*-th gene, $\mu_{j}$ and $\sigma_{j}$ are the mean and standard deviation for the *j*-th gene.

We define $M^{g}\in R^{m\times d^{g}}$ as the gene expression feature matrix, where *m* is the number of cancer cell lines, $d^{g}$ is the dimension of gene expression feature, and each line is the feature vector of a cell line. We adopt a late-integration approach in which each neural layer initially learns the features of specific omics feature and then aggregates them. We encode the gene expression feature of cell lines into an *f*-dimensional representation matrix $z_{g}$:

(3) $$z_{g}=M^{g}W,$$

where $z_{g}\in R^{m\times f}$, $W\in R^{d^{g}\times f}$ is the weight matrix of the linear transformation. Similarly, we have the $z_{c}\in R^{m\times f}$ as the copy number variation feature matrix.

After obtaining multiomic representations of cell lines, we assign different attention weights to the data to create a global reference during model training and prediction. This results in assigning higher weights to essential information and reducing the influence of less effective omics data with lower weights. Given the learned gene expression representation $z_{g}$ and the learned copy number variation representation $z_{c}$, in order to obtain the attention scores of different omics data, we first learn the weight scores of the two-omics representations $w_{g}$ and $w_{c}$ as follows:

(4) $$w_{g}=\tanh\left(W_{g}\cdot {\left(z_{g}\right)}^{T}+b\right),$$

(5) $$w_{c}=\tanh\left(W_{c}\cdot {\left(z_{c}\right)}^{T}+b\right),$$

(6) $$\alpha_{g}=\text{softmax}\left(w_{g}\right)=\frac{\;\text{exp}\;\left(w_{g}\right)}{\;\text{exp}\;\left(w_{g}\right)+\text{exp}\;\left(w_{c}\right)},$$

(7) $$\alpha_{c}=\text{softmax}\left(w_{c}\right)=\frac{\;\text{exp}\;\left(w_{c}\right)}{\;\text{exp}\;\left(w_{g}\right)+\text{exp}\;\left(w_{c}\right)},$$

where $W_{g}$ and $W_{c}\in R^{1\times f}$ are the learnable weigth matrices, *b* is the learnable bias vector. The weight scores $w_{g}$ and $w_{c}$ can be normalized by the softmax function to obtain the attention score $\alpha_{g}$ and $\alpha_{c}$. Two-omics representations $z_{g}$ and $z_{c}$ can be fused through attention:

(8) $$h_{\mathrm{omics}}=\alpha_{g}\cdot z_{g}+\alpha_{c}\cdot z_{c},$$

where $h_{\mathrm{omics}}\in R^{m\times f}$, which is the feature vector of aggregated multiomics data after the attention.

#### 2.2.2 Morphology image feature representation

We obtain morphological images for 254 cell lines from the GMDZ dataset and preprocess them into the jpg format. We normalize the input to scale all cell morphology images to a size of 224 × 224. To derive morphological representations of these cell lines from the images, we utilize a two-layer CNN with average pooling, yielding the representation denoted as $h_{\mathrm{image}}\in R^{m\times f}$. We finally obtain the representation of cells $Z^{\mathrm{cell}-\mathrm{line}}\in R^{m\times 2f}$ by concatenating multiomics representation and morphological image representation of cell lines:

(9) $$Z^{\mathrm{cell}-\mathrm{line}}=[h_{\mathrm{omics}};h_{\mathrm{image}}].$$

### 2.3 Drug molecular graph representation learning

PubChem contains a vast amount of chemical structure data for drugs. To learn drug representations, we collect the compound IDs of 311 drugs and their corresponding SMILES strings from PubChem. Next, we utilize the ConvMolFeaturizer method (Duvenaud *et al.* 2015) in DeepChem. This method converts the SMILES string of webeeach drug into a molecular graph, consisting of chemical atoms and the bonds that connect them. Each atom’s characteristics within the drug can be represented by a 75-dimensional feature vector. Consequently, we obtain the molecular graph and feature matrix associated with each drug, which will be used as input data for the GCN.

Then, a drug can be described as a molecular graph, with nodes representing chemical atoms and edges representing chemical bonds. For a given drug, we denote its molecular graph as $G^{d}=(X^{d},A^{d})$, where $X^{d}\in R^{n\times f^{d}}$ is the feature matrix for drug *d*, recording the features of each atom in the drug ($f^{d}=75$), and $A^{d}\in R^{n\times n}$ is the adjacency matrix representing the bonds. Here, *n* is the number of atoms in the molecular graph of drug *d*. We can then utilize GCN to learn representations for each node within a drug. The drug node representation, denoted as $Z^{\mathrm{drug}}$, can be calculated as follows:

(10) $$Z^{\mathrm{drug}}=\sigma \left({\tilde{D}}^{-\frac{1}{2}}{\tilde{A}}^{d}{\tilde{D}}^{-\frac{1}{2}}X^{d}W\right),$$

where ${\tilde{A}}^{d}=A^{d}+I$, $W\in R^{f^{d}\times 2f}$ is the learnable weight matrix, and $\tilde{D}$ is the graph diagonal degree matrix.

In our GCN model, we employ two consecutive GCN layers with the ReLU activation function applied after each GCN layer. Considering that different drugs have different numbers of atoms, we incorporate a global max-pooling layer after applying two GCN layers to capture the comprehensive drug graph representation. This pooling operation condenses an *n*-atom drug molecule graph into a 2*f*-dimensional vector. This representation is subsequently combined with the cell line representation to make a prediction for the CDR.

### 2.4 Cancer drug response prediction

With the previously obtained multiomics cell line representation, morphology image cell line representation, and drug molecular structure representation, we now move forward to predict CDR.

#### 2.4.1 Multimodal contrastive learning

We propose a contrastive learning approach for multiomics and morphology image cell line representations. The goal is to minimize variance within multimodal data by grouping samples from the same cell line together, while simultaneously maximizing the variance among different cell lines by separating them. This unsupervised method is designed to automatically align cell line representations derived from multimodal data. In the context of calculating the contrastive loss, it’s crucial to define positive and negative samples. In our training dataset, there are *i* cell lines, each comprising *N* pairs of cell line omics-images, which are explicitly distinguished during training:

(11) $$L_{o2I}=-\frac{1}{N}\sum_{i=1}^{N}\;\text{log}\;\frac{\;\text{exp}\;\left(h_{o\mathrm{mics}}{}^{⊤}h_{i\mathrm{mage}}/\gamma \right)}{\sum_{j=1}^{N}\;\text{exp}\;\left(h_{o\mathrm{mics}}{}^{⊤}h_{i\mathrm{mage}}/\gamma \right)},$$

(12) $$L_{I2o}=-\frac{1}{N}\sum_{i=1}^{N}\;\text{log}\;\frac{\;\text{exp}\;\left(h_{i\mathrm{mage}}{}^{⊤}h_{o\mathrm{mics}}/\gamma \right)}{\sum_{j=1}^{N}\;\text{exp}\;\left(h_{i\mathrm{mage}}{}^{⊤}h_{o\mathrm{mics}}/\gamma \right)},$$

(13) $$L_{cl}=\frac{1}{2}\left(L_{o2I}+L_{I2o}\right),$$

where γ is the temperature parameter, which is a hyperparameter.

#### 2.4.2 CDR prediction

We perform the CDR prediction based on the learned representations. Firstly, we construct cell line-drug pairs by combining the representations of cell lines and drugs as follows:

(14) $$Z_{mn}=[Z_{m}^{\mathrm{cell}-\mathrm{line}},Z_{n}^{\mathrm{drug}}],$$

where $Z_{mn}$ is the concatenation of the *m*-th cell line ($Z_{m}^{\mathrm{cell}-\mathrm{line}}$) and the *n*-th drug ($Z_{n}^{\mathrm{drug}}$). Then, we model the task as a binary classification task and train an MLP model for CDR prediction.

To minimize the difference between predicted CDR association probabilities and the ground truth, we utilize cross-entropy as the classification loss $L_{c}$:

(15) $$L_{c}=\sum_{i=1}^{S}\left(y_{i}\;\text{log}\;\left(p_{i}\right)+\left(1-y_{i}\right)\;\text{log}\;\left(1-p_{i}\right)\right),$$

where $y_{i}$ is the true label for the *i*-th cell line-drug pair (i∈S), and $p_{i}$ is the predicted probability of *i*-th cell line-drug pair.

Hence, the final loss function L of MMCL-CDR, which combines contrastive learning $L_{cl}$ and cross-entropy loss $L_{c}$, can be defined as follows:

(16) $$L=\alpha L_{c}+\beta L_{cl},$$

where α and β are the hyperparameters.

## 3 Experiments

In this section, we conduct experiments to address the following research questions (RQs): RQ1: Is it feasible and effective to predict cell line-drug associations using the proposed framework? RQ2: Is it beneficial to improve cell line representation learning through the contrastive learning of multiple omics data with morphology images of cell lines? RQ3: Do the aggregation methods employed by our model effectively consolidate information and enhance the CDR prediction accuracy?

### 3.1 Datasets

In this section, we will provide a comprehensive overview of our dataset. Our dataset is constructed from three publicly available resources: GDSC, PubChem, and DMSZ. Detailed statistics of the dataset are summarized in Table 1.

**Table 1.** Statistics of datasets.

Data type	Nodes	Features	Address
Drug SMILES	311 drugs	Drug molecular graph	https://pubchem.ncbi.nlm.nih.gov/
Genomics	254 cell lines	23 914 genes	https://www.cancerrxgene.org/
Transcriptomics	254 cell lines—23 914 genes	23 914 genes	https://www.cancerrxgene.org/
Morphology image	254 cell lines	254 morphology images	https://www.dsmz.de/

**GDSC** is a significant resource for the discovery of therapeutic biomarkers in cancer cells, offering access to a vast collection of over 1000 distinct cancer cell lines. These cell lines have been meticulously chosen to comprehensively represent the spectrum of both common and rare cancer types affecting adults and children, encompassing various cell origins, such as epithelial, mesenchymal, and hematopoietic cells.

**PubChem** serves as a widely used chemical information resource with various applications in the medical field. It comprises three interconnected databases: Substance, Compounds, and Bioassays. The compound database, which is employed in this article, contains distinct chemical structures extracted from the Substance database.

**DMSZ** is instrumental for the future advancement of science, public health and bioeconomy. With its comprehensive collection of biological material and its unique expertise in the fields of cultivation, identification, taxonomy/phylogeny and conservation. DSMZ also plays a key role in biodiversity applications.

### 3.2 Experimental settings

#### 3.2.1 Baselines

We select a set of baselines for comprehensive comparison of MMCL-CDR:

- **DeepDSC** (Li *et al.* 2021) utilizes deep autoencoder to obtain the low-dimensional representation of cell lines and integrate the molecular characteristics of drugs into this model to predict the sensitive data of cell line drugs
- **GraphCDR** (Liu *et al.* 2022a) constructs a graph neural network of cell line multiomics data and drug molecular structure for CDR prediction, while using contrastive learning to improve the generalization ability of the model.
- **NIHGCN** (Peng *et al.* 2022) proposes a heterogeneous graph convolutional network based on neighborhood interaction, which considers the neighbor interaction in the graph neural network, and predicts drug response through the linear correlation coefficient of features.
- **GraphDRP** (Nguyen *et al.* 2022) represents drugs directly as molecular graphs, while cell lines are described as binary vectors. The characteristics of drugs and cell lines are learned through convolutional layers. Finally, a fully connected neural network is used to predict the response value of each cell line-drug pair.

#### 3.2.2 Evaluation metrics

We employ two evaluation metrics: **AUC** (area under the curve) and **AUPR** (area under the precision-recall curve), consistent with previous studies.

The **ROC** (receiver operating characteristic) curve plots the true positive rate on the *y*-axis against the false positive rate on the *x*-axis, serving as an evaluation tool for evaluating a model’s accuracy. The area under the ROC curve, denoted as **AUC**, is a widely used statistic in scientific research for evaluating binary classification models. Its value ranges from 0 to 1, and **AUC** represents the area under the ROC curve. Importantly, **AUC** is not influenced by the distribution of positive and negative samples, making it a robust metric for model assessment.

**AUPR** is a metric that quantifies the AUPR. In this curve, the *x*-axis represents recall, and the *y*-axis represents precision. AUPR provides an intuitive measure for evaluating a model’s performance. When both **AUC** and **AUPR** values are closer to 1, it indicates better model performance, with higher precision and recall.

#### 3.2.3 Experimental setup

We have obtained 7809 sensitive pairs and 14 681 resistant pairs as our dataset, where sensitive pairs represent positive examples, while resistant pairs serve as negative examples. For MMCL-CDR, we set the embedding dimension for both cell lines and drugs to 36, and the hyperparameter γ is set to 0.01. The model is trained using the Adam optimizer (Zhang 2018) for 2000 epochs, with a learning rate of 0.008 and a weight decay of 1e−5. The hyperparameters of the loss function part, α and β are set to 0.6 and 0.4, respectively. We have employed a 10-fold cross-validation procedure, where all cell lines and drug response pairs were divided into 10 subsets. In each iteration, nine of these subsets were selected as the training set, while the remaining one was designated as the test set. This process ensures a thorough evaluation of the model’s performance across different data splits. For all the baseline methods, we replicate their experiments on our dataset with the same experimental settings and model parameters as described in their papers.

### 3.3 Experiment results

#### 3.3.1 CDR prediction

We compare our model with four baseline methods in the task of compound-drug response (CDR) prediction. The detailed AUC and AUPR results are presented in Table 2. When compared with the other methods, the experimental results demonstrate that our model achieves the highest performance in predicting cell line-drug associations. Notably, our model exhibits at least a 1.5% improvement in both AUC and AUPR compared to the most competitive baseline, GraphDRP.

**Table 2.** The comparison results of MMCL-CDR and baseline methods.

Method	AUC	AUPR
DeepDSC	0.7592	0.7733
GraphCDR	0.8153	0.8428
NIHGCN	0.8642	0.8534
GraphDRP	0.8754	0.8875
MMCL-CDR	**0.8890**	**0.9001**

Bold values show the best performance for each criterion.

**Figure 2.**
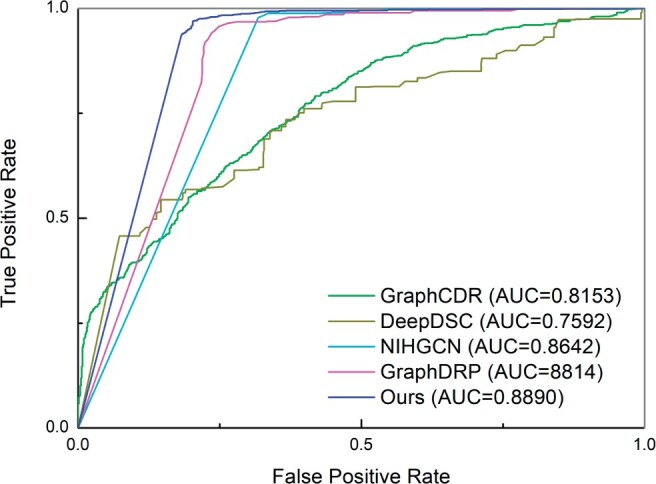
ROC curves of our method MMCL-CDR and baseline methods.

Figure 2 presents the ROC curves for our model and other state-of-the-art methods in CDR prediction. Our model consistently demonstrates a higher ROC curve in comparison with the other methods, highlighting the effectiveness of our model in automatically learning cell line and drug representations that lead to accurate cancer drug response prediction.

#### 3.3.2 Ablation study

As our model integrates the multiomics and multimodal representation of cell lines, we conduct ablation experiments to validate its effectiveness. In Table 3, “w/o morphology image” means our model without morphology image of cell lines, “w/o RNA-Seq” represents our model without gene expression of cell lines, “w/o CNV” represents our model without copy number variations of cell lines, “w/o CNV, morphology image” represents the model only uses gene expression of cell lines, “w/o RNA-Seq, morphology image” represents the model only uses copy number variations of cell lines, and “w/o CNV, RNA-Seq” represents the model only uses morphology image of cell lines.

**Table 3.** The influence of different cell line omics data on the results.

Omics-data	AUC	AUPR
w/o morphology image	0.8187	0.8529
w/o RNA-Seq	0.8853	0.8981
w/o CNV	0.8703	0.8842
w/o CNV, morphology image	0.8292	0.8646
w/o RNA-Seq, morphology image	0.8201	0.8584
w/o CNV, RNA-Seq	0.8252	0.8624
Full model	**0.8890**	**0.9001**

From the results in Table 3, we can draw the following conclusions: The full model, which integrates multiomics and multimodal information of cell lines (RNA-Seq, CNV, and cell line morphology images), achieves the best performance in terms of AUC and AUPR metrics. Excluding the cell line morphology images from the model leads to a noticeable decrease in AUC and AUPR values, and the performance of only using morphological image data is better than using CNV data, highlighting the importance of including morphological information in the model. Morphological information can provide important insights into the phenotypic changes caused by drugs, which can help predict their potential therapeutic effects and side effects. The exclusion of RNA-seq data also results in a noticeable decrease in AUC and AUPR values, showing that RNA-seq data can provide insights into the transcriptional changes caused by drugs, which are important indicators of drug response and disease progression. The exclusion of CNV data leads to a relatively smaller decrease in AUC and AUPR values compared to excluding RNA-seq or cell line morphology images, indicating that copy number variations may not be as critical as transcriptomic or morphological features for predicting CDR. However, CNV data can still provide valuable information about genetic mutations and aberrations that may affect drug response, especially for targeted therapies. The decrease in AUC and AUPR values when excluding multiple modalities supports the hypothesis that integrating multiple omics datasets provides complementary information that enhances the performance of the model. This finding is consistent with previous studies.

Our model utilizes both an attention mechanism and contrastive learning to update the representations of cell lines. To verify the role of these two modules, we conduct additional ablation experiments to compare our model with three variants: MMCL-CDR without attention “w/o Attention,” MMCL-CDR without contrastive learning “w/o CL,” and MMCL-CDR without both attention and contrastive learning “w/o CL and Attention.” From the results in Table 4, we can draw the following conclusions: The full model, which integrates attention mechanism and contrastive learning, achieves the best performance in terms of AUC and AUPR metrics. Removing attention or contrastive learning leads to a decrease in performance, highlighting the importance of both methods for the model. When contrastive learning and attention mechanism are removed simultaneously, the performance of the model will drop by more than when only one of the methods is removed, indicating that the interaction between the two methods is synergistic and their combined use is more effective for CDR prediction. The experimental results show that our model’s performance is maximized when both attention mechanism and contrastive learning are used together.

**Table 4.** The comparison results of MMCL-CDR and its degeneration models.

Model	AUC	AUPR
w/o Attention	0.8843	0.8872
w/o CL	0.8700	0.8914
w/o CL and attention	0.8563	0.8743
Full model	**0.8890**	**0.9001**

#### 3.3.3 Parameter sensitivity analysis

**Figure 3.**
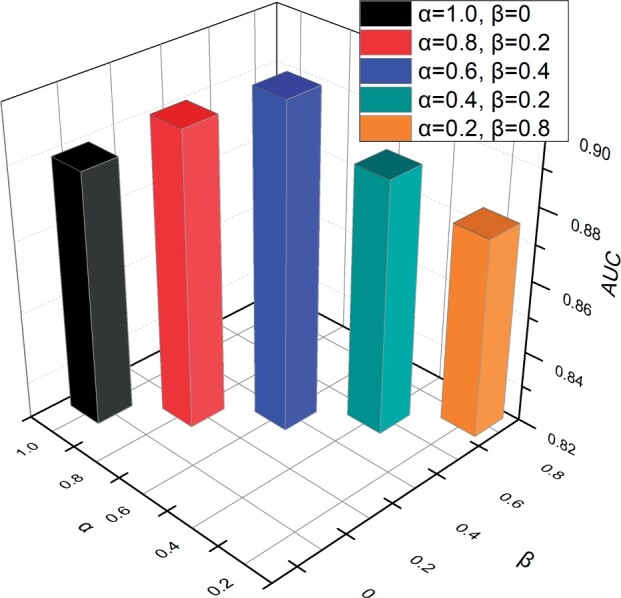
The results for varying hyperparameters α and β.

We conduct sensitivity analysis on our model to evaluate its robustness. We analyze the influence of the hyperparameters α and β of contrastive learning $L_{cl}$ and cross-entropy loss $L_{c}$ on the experimental results. The different experimental results are shown in Fig. 3. In the case of the MMCL-CDR loss function L, where the hyperparameters α+β=1, we systematically decrease the value of α while increasing the value of β, and examine the impact of these hyperparameters on the experimental outcomes. Here, α=1 represents “without contrastive learning.” As shown in Fig. 3, the best results are achieved when α was set to 0.6. Other values lead to a decrease in the experimental results.

#### 3.3.4 Qualitative analysis

**Figure 4.**
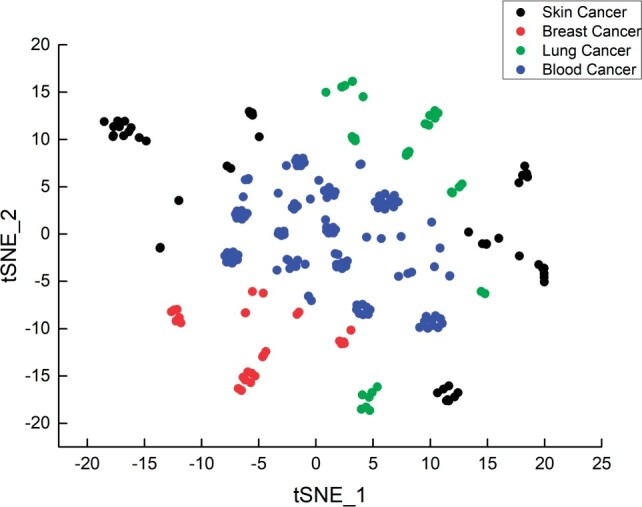
Visualization of cancer cell lines.

To better observe the cell line representation learned by MMCL-CDR, we apply t-Distributed Stochastic Neighbor Embedding (t-SNE) (Van der Maaten and Hinton 2008), which can visualize high-dimensional data as a two- or three-dimensional map. We randomly selected four types of cancer cell lines (skin cancer, breast cancer, lung cancer, and blood cancer) for visualization. We first processed the high-dimensional cell line representation into a two-dimensional representation using t-SNE. Then different types of cancer cell lines were labeled with different colors. The distribution of the cancer cell line representations can be observed in Fig. 4. Cancer cell lines of the same class are clustered closer together, indicating that MMCL-CDR can identify good representations of different cancer cell lines.

**Figure 5.**
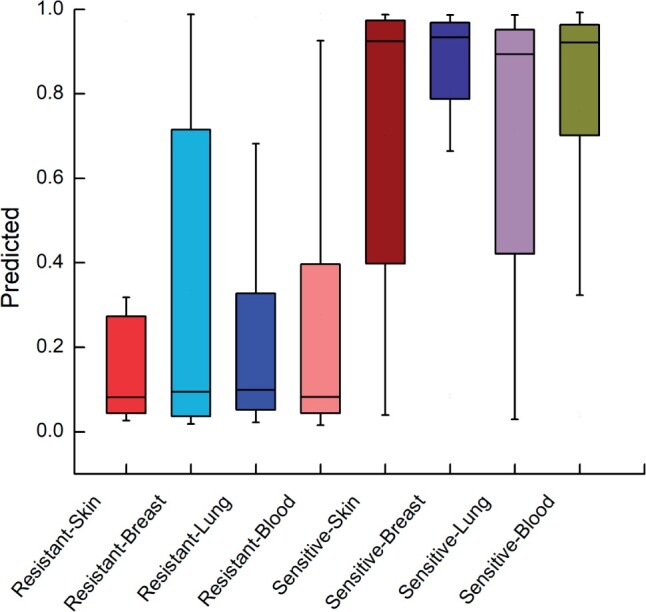
Box plot of different classes of cancer cell lines.

We also plotted a box chart for drug sensitivity and drug resistance of four types of cancers separately. The prediction results for different drug responses are presented in Fig. 5. It can be observed that our model demonstrates good classification performance for both kinds of drug responses.

#### 3.3.5 Case study

We conduct a case study of several resistant drugs of the cancer cell line *A498* based our model. Detailed results are shown in Table 5.

**Table 5.** Cancer drug response prediction results on cell line *A498*.

Cell line	Drug	Prediction result	Ground-truth
*A498*	A-770041 Avagacestat AS601245 BI-2536 Cabozantinib Etoposide NSC-87877 5-Fluorouracil	Resistant Resistant Resistant Resistant Resistant Resistant Resistant Sensitive	Resistant Resistant Resistant Resistant Resistant Resistant Resistant Resistant

We find MMCL-CDR can accurately classify the majority of drugs that are resistant to *A498*. Our model made a sensitive prediction for *5-Fluorouracil* in the *A498* cell line. While, the GDSC database reports that the ${\text{IC}}_{50}$ value of the drug *5-Fluorouracil* in the cell line *A498* is 566.28. This value exceeds the drug’s maximum screening concentration value of 32.0. Hence, the response of the drug *5-Fluorouracil* to the *A498* cell line should be classified as resistant according to the “Ground-truth.” However, there is a notable decrease in cell viability observed at all concentrations of *5-Fluorouracil*, and the substantial impact of *5-Fluorouracil* on the *A498* cell line has been validated by other researchers (Kim *et al.* 2020). This is consistent with our model’s prediction of drug sensitivity in the *A498* cell line, indicating that our model has the potential to discover new anticancer drugs.

## 4 Conclusion

In this study, we proposed a model, MMCL-CDR, for predicting cancer drug response using multiomics and morphology images with contrastive learning. Through comprehensive experiments, we proved that MMCL-CDR can achieve state-of-the-art results in CDR prediction. MMCL-CDR overcomes the lack of multiomics and multimodal data in most existing research and can comprehensively capture multiple potential interactions between cell lines and drugs. MMCL-CDR discovered the causal relationships between cell lines and drugs through attention mechanism, and for the first time, explored contrastive learning of cell line morphological images and multiomics data, providing a novel perspective for drug response prediction research. It is worth mentioning that although our model mainly utilizes two kinds of omics data, it can also be extended to other omics data such as proteomics (Hanash 2003, Meissner *et al.* 2022) and radiomics (Kumar *et al.* 2012). By accurately predicting drug sensitivity, our model can serve as a valuable tool for researchers, facilitating the discovery and development of novel anticancer drugs. This potential contribution has the capacity to accelerate advancements in cancer treatment, ultimately leading to improved patient outcomes.

## Data Availability

The source code and dataset of DeepITEH have been uploaded to https://github.com/catly/MMCL-CDR.
